# Microbiological Assessment of Dairy Products Produced by Small-Scale Dairy Producers in Serbia

**DOI:** 10.3390/foods13101456

**Published:** 2024-05-08

**Authors:** Biljana Aleksic, Bozidar Udovicki, Jovana Kovacevic, Zorana Miloradovic, Ilija Djekic, Jelena Miocinovic, Nikola Tomic, Nada Smigic

**Affiliations:** 1 Department of Food Safety and Quality Management, Faculty of Agriculture, University of Belgrade, Nemanjina 6, 11080 Belgrade, Serbia; biljana1972aleksic@gmail.com (B.A.); bozidar.udovicki@agrif.bg.ac.rs (B.U.); idjekic@agrif.bg.ac.rs (I.D.); tsnikola@agrif.bg.ac.rs (N.T.); 2 Dairy Institute, Smolucska 11, 11070 Belgrade, Serbia; 3 Food Innovation Center, Oregon State University, 1207 NW Naito Parkway, Portland, OR 97209, USA; jovana.kovacevic@oregonstate.edu; 4 Department of Animal Source Food Technology, Faculty of Agriculture, University of Belgrade, Nemanjina 6, 11080 Belgrade, Serbia; zorana@agrif.bg.ac.rs (Z.M.); jmiocin@agrif.bg.ac.rs (J.M.)

**Keywords:** raw milk cheese, pasteurized milk cheese, kajmak, open market, *Listeria monocytogenes*

## Abstract

The microbiological quality of dairy products from small-scale producers in Serbia was analysed. A total of 302 dairy products [raw (*n* = 111) and pasteurized milk cheeses (*n* = 79) and kajmak (*n* = 112)], were collected and tested for the presence of pathogens, *Listeria monocytogenes* and *Salmonella* spp., and enumerated for Coagulase-positive staphylococci (CPS), *Escherichia coli*, and yeasts and moulds. None of the samples tested positive for *Salmonella* spp., while *L. monocytogenes* was recovered from one raw milk cheese and five kajmak samples. Raw milk cheese and kajmak also had higher levels of indicator microorganisms, namely *E. coli* and yeast and moulds. Molecular serotyping grouped *L. monocytogenes* isolates into serogroups 1 (1/2a and 3a) and 3 (1/2b, 3b, and 7). When exposed to eight antibiotics, *L. monocytogenes* isolates were mostly sensitive, with the exception of oxacillin and reduced susceptibility to clindamycin, penicillin G, and trimethoprim/sulfamethoxazole, emphasizing the importance of continuous surveillance for antimicrobial resistance. Samples that tested positive for *Listeria* spp. also had higher loads of indicator microorganisms, namely *E. coli* and yeast and moulds, suggesting lapses in hygiene practices during production. Collectively, these data emphasize the need for improved food safety and hygiene practices among small-scale dairy producers. This is crucial to reduce the microbial contamination and improve both the quality and safety of dairy products in the Serbian market.

## 1. Introduction

The Republic of Serbia has a long tradition of dairy products that reflects its cultural heritage. This tradition is rooted in the wisdom and experience of producers and has been passed down through generations [1,2]. Among dairy products, white brined cheeses (WBCs) and kajmak are most popular [1,3], most often sold at open markets [4]. Unlike street stalls or occasional food fairs, these markets offer a consistent supply of different dairy products, encouraging community interaction [1,3]. Open markets typically serve as dynamic centres for preserving culinary traditions and connecting producers and consumers.

Traditional cheese production in Serbia takes place predominantly in small-scale dairy operations that use either raw or pasteurized milk [5]. In general, northern Serbia is known for the production of raw milk cheeses [6], while, in the central and eastern parts of the country, such cheeses are mostly produced from pasteurized milk [7,8]. Traditional cheese production does not use starter cultures but relies on the natural microbiota, while coagulation is achieved by adding rennet [5,9]. In western Serbia, kajmak is produced by separating the aggregated milk fat on the surface of boiled milk. The milk remaining after kajmak production is partially skimmed and serves as a raw material for the production of semi-fat WBC [10]. Apart from geographical differences, there are also differences in traditional production within individual households [11]. The innate lactic acid bacteria present in raw milk [5] and the extensive environmental microbiota [12] have a significant influence on these methods. Consequently, differences in the sensory, microbiological, and overall quality of the final products are evident [13,14].

The composition of raw milk and dairy products provides a favourable environment for the proliferation of pathogenic and spoilage microorganisms [15,16]. Food safety and quality risks have been identified in the context of traditional production and distribution, often associated with open markets [17,18]. Outbreaks related to dairy products, including cheese, are often associated with specific microorganisms. In particular, these include *Listeria monocytogenes*, *Escherichia coli* O157:H7, *Salmonella* spp., *Staphylococcus aureus*, *Brucella* spp., *Clostridium botulinum,* and *Campylobacter jejuni* [19,20,21]. Notably, foodborne outbreaks linked to cheeses are typically associated with soft or raw milk cheeses [22].

*L. monocytogenes* is considered one of the most prominent pathogens associated with ready-to-eat foods (RTE), including a number of cheeses [23]. Its presence throughout the food supply [24] is of particular concern due to high mortality rates associated with invasive listeriosis [25]. Vulnerability to infection is especially pronounced in immunocompromised individuals, the elderly, newborns, and pregnant women [26]. While pasteurization is effective at inactivating the initial pathogen load in raw milk [27,28], the presence of *L. monocytogenes* in dairy processing facilities poses a risk due to possible post-pasteurization contamination [29]. Most recently, a multi-state outbreak of listeriosis in the United States was linked to the consumption of fresh, Hispanic-style soft cheeses made from pasteurized milk [30]. The pathogen is considered sensitive to most antibiotics used in the treatment of infections caused by Gram-positive bacteria [31], except for older quinolones, cephalosporins, and fosfomycin, for which innate resistance has been identified [32]. However, in the last decade, changes in the incidence and type of antimicrobial resistance in foodborne *L. monocytogenes* strains has been reported [33]. The over-prescription of antibiotics in clinical practice and their intensive use for prophylactic purposes in animal husbandry are considered important factors in the development of bacterial resistance to antibiotics [34].

Although *Salmonella* spp. are most commonly found in RTE meat and meat products, spices, and herbs [35], these pathogens have also been implicated in outbreaks associated with the consumption of cheese [36]. *Salmonella* spp. colonize the intestinal tract of warm-blooded animals, including cattle, and are commonly found in raw milk and dairy products made from raw milk [37]. A review by Gould et al. [20] reported that *Salmonella* spp. was the most frequently isolated pathogen associated with dairy products, with the highest prevalence in raw milk cheese (34%). Consumption of raw milk cheese has also been associated with outbreaks caused by rare strains of this pathogenic bacterium [38,39,40] and with very low infectious doses [36].

Coagulase-positive staphylococci (*Staphylococcus aureus*, *S. intermidius*, *S. hyicus,* and others) (CPS) have been associated with animal diseases such as mastitis [37] but also with food poisoning in humans [41]. This occurs when people consume food containing one or more previously formed enterotoxins [42]. When the number of CPS in cheese exceeds 5 log CFU/g, this can lead to the potential formation of highly heat-resistant staphylococcal enterotoxins [43]. Dairy products are often implicated in cases of food poisoning due to the presence of enterotoxins [41,44]. CPS are found primarily in raw milk [45] and products derived from it [22,46], establishing raw milk as the main source of introduction of CPS into the dairy processing chain [47]. In contrast, the presence of CPS in thermally treated dairy products is often due to human recontamination [48] or poor hygiene practices in dairy processing facilities [49].

*Escherichia coli*, a bacterium that commonly inhabits the intestines of humans and warm-blooded animals, is widely considered as an indicator of faecal contamination [50]. Its presence in raw milk and cheese is closely associated with poor hygiene practices [51]. While many *E. coli* strains are non-pathogenic, certain groups within the genus [Verocytotoxin-producing *E. coli* (VTEC), especially strain *E. coli* O157:H7, enterotoxigenic *E. coli* (ETEC), enteroinvasive *E. coli* (EIEC), enteropathogenic *E. coli* (EPEC), enteroaggregative *E. coli* (EAggEC), and diffusely adherent *E. coli* (DAEC)] are known causative agents of foodborne outbreaks [22,52,53].

In addition to bacteria, yeasts and moulds play a significant role in the dairy industry. In certain cheeses, yeasts contribute to the development of different sensory characteristics by fermenting lactose, utilizing lactic acid, and participating in lipolytic and proteolytic activities [54]. However, an excessive growth may lead to cheese spoilage causing undesirable effects such as gas formation, off flavours, discoloration, and texture alterations [55]. A study by Rogga et al. [56] also reported that spoilage yeasts may play a protective role in the survival of *L. monocytogenes* by raising pH through their proteolytic activity. Although moulds are inevitable in the production of some cheese varieties [57], they can affect both food quality and food safety [58].

While previous studies have examined specific groups of microorganisms in Serbian dairy products, such as *Enterobacteriaceae* [59], *Staphylococcus aureus* [2], enterococci, *Micrococaceae*, yeasts and moulds [11], and lactic acid bacteria [60,61,62], not many have looked at the broader range of microorganisms or included *L. monocytogenes* or *Salmonella* spp. By including pathogens such as *L. monocytogenes* and *Salmonella* spp., as well as hygiene indicators such as CPS, *E. coli*, and yeasts and moulds, this study aimed to provide a more holistic understanding of the microbiological quality of dairy products produced by small-scale dairy producers available in Serbia.

## 2. Materials and Methods

### 2.1. Sample Collection

This study investigated the quality and safety of dairy products originating from small producers in Serbia. These producers are defined in the Serbian regulations as “households” if they process up to 200 L of raw milk per week from their own farm and sell it locally or “small dairies” if they process up to 5000 L of raw milk per week and are approved to sell throughout Serbia [63]. Thirty open markets were visited in 18 cities, nine of which were in Belgrade, three in Zrenjanin, and three in Novi Sad. In the remaining 15 cities, there was one market visited in each, covering different regions of Serbia. Each market was visited once, targeting the purchasing of products from different producers. Depending on availability, various dairy products such as raw milk cheese, pasteurized milk cheese, and kajmak were purchased. Sampling took place mainly on weekends in order to reach a larger number of sellers. In addition, samples produced by small dairies were purchased from six retail outlets in Belgrade that specialize in such products. This approach allowed us to achieve comprehensive sampling in different regions and different open markets.

Overall, 302 dairy products (79 raw milk cheeses; 111 pasteurized milk cheeses; and 112 kajmak samples) made from cow’s milk were obtained in 2020–2021 and 2023 (Table 1). Our sampling approach targeted approximately 100 products in each category of raw milk cheese, pasteurized milk cheese, and kajmak, which are representative dairy products made by the small-scale producers. Samples were collected during late spring to the end of summer, over several years, including 2020, 2021, and 2023. Specifically, from May 2020 to August 2021, 198 samples were purchased from open markets and retail stores, including 40 raw milk cheeses, 100 pasteurized milk cheeses, and 58 kajmak samples. From March to July 2023, an additional 104 samples were purchased directly from open markets, including 39 raw milk cheeses, 11 pasteurized milk cheeses, and 54 kajmak samples. While we targeted up to 100 samples in each category, some categories ended up with fewer and some with more samples, due to the assortment and availability of dairy products during the time of purchase.

The samples (250 g) were purchased, packaged in PVC containers or plastic bags, labelled, and transported to the laboratory in Styrofoam boxes with reusable ice packs. Samples were analysed within 24 h of collection. Sample information was obtained by interviewing the sellers/producers at the site of purchase or from the product labels for the dairy products purchased at retail stores. This included information such as the type of milk used for the production (raw or pasteurized) and the degree of product ripeness. The microbiological characteristics of all products were analysed in an accredited laboratory of the Dairy Institute, Belgrade, Serbia [64].

### 2.2. Microbiological Analysis

The microbiological quality of the dairy products (79 raw milk cheeses; 111 pasteurized milk cheeses; and 112 kajmak samples) was assessed using the standard microbiological methods. The detection of *Salmonella* spp., *Listeria monocytogenes,* and *Listeria* spp. was conducted in accordance with ISO methods [65,66]. Hygiene indicators and spoilage microorganisms (CPS, *Escherichia coli*, and yeasts and moulds) were analysed using the ISO methods [67,68,69].

The detection of *Salmonella* spp. was performed according to the ISO 6579-1:2017 method [65]. Briefly, 25 g of the sample was diluted and homogenously mixed with 225 mL of buffered peptone water (BPW, 0.5% w/v NaCl, Himedia, Mumbai, India) and incubated at 37 °C for 18 h [65,70]. For selective enrichment, Rappaport–Vassiliadis medium with soya (RVS broth, Himedia, Mumbai, India) and Muller–Kauffman tetrathionate broth with novobiocin (MKTTn broth, Himedia, Mumbai, India) were inoculated and incubated at 41.5 °C and 37 °C, respectively. Following the incubation period, samples were plated by streaking onto two selective solid media, Xylose–Lysine Deoxycholate agar (XLD; Himedia, Mumbai, India) and Brilliant–green agar (BG; Torlak, Belgrade, Serbia), and incubated at 37 °C for 24 h. Presumptive colonies were confirmed with biochemical tests.

The detection of *L. monocytogenes* and *Listeria* spp. was performed according to the ISO 11290-1:2017 method [66]. Briefly, 25 g of the sample was inoculated in 225 mL of selective liquid enrichment medium with a reduced concentration of selective agents (modified Fraser broth, Himedia, Mumbai, India), homogenized for 2 min, and incubated at 30 °C for 25 h. Following incubation, an aliquot (0.1 mL) was transferred from the modified Fraser broth to Fraser broth (Himedia, Mumbai, India) and incubated at 37 °C for 24 h. Samples were then plated by streaking onto two selective media, agar *Listeria* according to Ottaviani and Agosti (ALOA; Himedia, Mumbai, India) and PALCAM (Himedia, Mumbai, India), and incubated at 37 °C for 24 h. Colonies presumed to be *L. monocytogenes* and *Listeria* spp. Were further confirmed by Gram staining, catalase reaction, motility at 25 °C, the CAMP test, and fermentation of sugars (D-glucose, rhamnose, and xylose). Species identification from single colonies was performed at INSTITUT SUPERLAB (Belgrade, Serbia) using VITEK MS (bioMérieux, Marcy l’Etoile, France) according to the manufacturer’s instructions.

Hygiene and spoilage indicators were analysed by using quantitative microbiological methods. The limit of quantification was 1 log CFU/g [71]. The initial suspension was prepared by homogenously diluting 20 g of the sample in 180 mL of prewarmed sodium citrate solution (Himedia, Mumbai, India) for 2 min. Further dilutions were prepared in peptone salt solution (Biolab, Budapest, Hungary) [72]. One millilitre of the appropriate dilution was inoculated into two sterile empty Petri dishes using the pour plate technique. Triptone bile glucuronid agar (Biolab, Budapest, Hungary) and Sabourand dextrose chloramphenicol (Biolab, Budapest, Hungary) agar were used for the enumeration of *E. coli* and yeasts and moulds and incubated at 44 °C and 25 °C for 20 h and 5 days, respectively [68,69].

For the enumeration of CPS, 1 mL of the three successive inoculation levels was plated on to the surface of Baird Parker agar (Biolab, Budapest, Hungary). After the incubation period (37 °C, up to 48 h), colonies presumed to be CPS were transferred to brain heart infusion broth (BHI broth, Biolab, Budapest, Hungary) and incubated at 37 °C for 24 h. The confirmation was performed by a tube rabbit plasma coagulase test [67].

In accordance with ISO methods [65,66], results for the *L. monocytogenes*, *Listeria* spp., and *Salmonella* spp. analysis were reported as detected/not detected in 25 g. For the analysis that included enumeration, the total number of the target microorganisms was reported as specified in the ISO methods [67,68,69,71] and converted to log CFU/g.

### 2.3. Serotyping of L. monocytogenes Isolates

Molecular serotyping of *L. monocytogenes* isolates (*n* = 6) was performed following a previously described method [73]. The multiplex PCR mixture (25 μL) consisted of 1 Unit of Taq DNA polymerase; 1X PCR buffer mix; 200 μM dNTPs (Thermo Fisher Scientific Baltics, UAB, Vilnius, Lithuania); 1 μM of each primer for lmo0737, ORF2819, and ORF2110; 1.5 μM of each primer for lmo1118; and 0.2 μM of each primer for prs. All primers were obtained from Integrated DNA Technologies (Leuven, Belgium) (Table 1).

**Table 1:** PCR target genes, primer sequences, and amplicon size for the serogrouping of *L. monocytogenes* isolates [73].

Target	Primer Sequence	Product Size (bp)	Serogroups
*prs*	F: GCTGAAGAGATTGCGAAAGAAG	370	*Listeria* spp.
	R: CAAAGAAACCTTGGATTTGCGG		
*lmo0737*	F: AGG GCT TCA AGG ACT TAC CC	691	*L. monocytogenes* 1/2a, 1/2c, 3a, and 3c
	R: ACG ATT TCT GCT TGC CAT TC		
*lmo1118*	F: AGG GGT CTT AAA TCC TGG AA	906	*L. monocytogenes* 1/2c and 3c
	R: CGG CTT GTT CGG CAT ACT TA		
*orf2819*	F: AGC AAA ATG CCA AAA CTC GT	471	*L. monocytogenes* 1/2b, 3b, 4b, 4d, and 4e
	R: CAT CAC TAA AGC CTC CCA TTG		
*orf2110*	F: AGT GGA CAA TTG ATT GGT GAA	597	*L. monocytogenes* 4b, 4d, and 4e
	R: CAT CCA TCC CTT ACT TTG GAC		

PCR was performed (Applied Biosystems 7500 PCR System, Foster city, USA) with an initial denaturation step at 94 °C for 4 min; 35 cycles of denaturation at 94 °C for 24 s, annealing at 53 °C for 75 s, and extension at 72 °C for 75 s; and a final incubation at 72 °C for 7 min. PCR amplification products (10 μL) were separated on a 2% UltraPure™ agarose gel (Serva, Heidelberg, Germany) in TBE buffer and stained with Serva DNA stain G (Serva, Heidlberg, Germany), run for 75 min at 110 V (OWL Easy cast TM B2, Thermo Scientific, Waltham, MA, USA). PCR products were visualized using UV transilluminator. A 1 Kb DNA ladder (Thermo Fisher Scientific Baltics, UAB, Vilnius, Lithuania) served as the size standard for each gel. *L. monocytogenes* ATCC 19111 and ATCC 13932 were used as controls.

### 2.4. Antimicrobial Resistance Screening of L. monocytogenes Isolates

The resistance of *L. monocytogenes* isolates to eight antibiotics (cefotaxime, CTA; clindamycin, CLI; gentamicin, GEN; oxacillin, OXA; penicillin G, PEN; tetracycline, TET; trimethoprim with sulfamethoxazole, TRS; and vancomycin, VAN) was tested using the disc diffusion method according to the Clinical and Laboratory Standards Institute (CLSI) guidelines [74,75]. The isolates were streaked on tryptone soy agar with yeast extract (TSAYE, HiMedia, Mumbai, India) and incubated at 37 °C for 24 h. A suspension (0.5 McFarland; DEN-1, Biosan, Riga, Latvia) from a single colony was transferred to Mueller-Hinton agar with 5% defibrinated sheep blood [76,77], and four antibiotic discs (Bioanalyse, Ankara, Turkey) were placed on the agar surface. Plates were incubated at 35 °C for 24 h. The resulting inhibition zones were measured (mm), classifying strains as resistant, intermediate, and sensitive. The results of the antimicrobial susceptibility tests were evaluated according to the susceptibility criteria set by the CLSI for *Staphylococcus* spp. and EUCAST for *L. monocytogenes* [74,75,78].

### 2.5. Data Analysis

For microbiological analyses that included enumeration, CFU/g values are provided, whereas for presence/absence analyses and where microbial counts were below the level of detection, the number of samples is reported.

For the calculation of statistical indicators (mean, standard deviation, and range), only samples with values above the limit of quantification were included. Fisher’s exact test was used to determine whether the microbiological quality of the different types of dairy products differed in terms of the hygiene and spoilage indicators depending on the type of milk from which the cheeses was made and the maturity level. The criteria for the microbiological quality of the dairy products were based on Serbian regulations [79,80], current recommendations [81], and literature data [52,55], as shown in Table 2. Following the evaluation (satisfactory, acceptable, or unsatisfactory), the relative frequencies within the observed product type were calculated and compared using Fisher’s exact test to assess the influence of the type of milk used for the cheese production and the ripeness of kajmak samples for each microbiological characteristic studied. The overall microbiological quality of a sample was rated as “unsatisfactory” if at least one individual result was unsatisfactory, in relation to the food safety and hygiene criteria presented in Table 2. A sample that had at least one microbiological result of acceptable and all others as satisfactory was given a rating of “acceptable” microbiological quality. Finally, a sample was rated as “satisfactory” if all microbiological test results were satisfactory.

Statistical analyses were performed using SPSS (version 21.0; IBM Corporation, Armonk, NY, USA) and Excel (version 2403; 2024, Microsoft Corp., Redmond, WA, USA). Results were considered significant at *p* < 0.05.

## 3. Results and Discussion

Microbiological quality assessment of traditional dairy foods produced by small-scale processors can be useful in providing the information on hygiene conditions during production and post-processing handling, as well as the potential food safety risks to consumers [83,84]. Here, we report the findings from the microbiological quality analyses of 302 artisanal dairy products collected at open markets and retail stores in Serbia.

### 3.1. The Presence of Pathogens in Dairy Products

None of the cheese and kajmak samples tested positive for *Salmonella* spp. The low prevalence of this pathogen in dairy products found in this study is consistent with a previous study in south-east Serbia, which found no *Salmonella* spp. in artisanal raw milk cheeses [59]. Studies of traditionally produced cheeses in Brazil [85], Italy [23], Poland [86], Canada [87], and the United Kingdom [52] have also reported the absence of *Salmonella* spp. in traditionally made products, while 36% of samples from open markets and local stores in Hungary harboured high levels of *Salmonella* spp. [88]. While raw milk cheeses represent a considerable risk for this pathogen [89], in rare cases, pasteurized milk can also be contaminated [90]. This emphasizes the need for robust hygiene and manufacturing practices when making traditional and artisanal cheeses and dairy products. It is noteworthy, however, that in our study, no evidence of *Salmonella* spp. was found either in raw milk cheese or in cheese made from pasteurized milk, indicating adequate control for this pathogen in the ingredients and/or production process.

In the current study, only one raw milk cheese (1.26%) was positive for *L. monocytogenes*, whereas 4.5% (*n* = 5) of kajmak samples harboured the pathogen (Table 3). While illnesses and listeriosis outbreaks have been linked to raw milk cheeses, in regions such as Brazil, Slovenia, Italy, and Poland [85,86,91,92], studies have also reported a general absence of *L. monocytogenes* in raw milk cheeses and only sporadic contamination was seen in the UK [52]. The primary route for pathogen introduction into dairies appears to be via contaminated raw milk [93]. However, the contamination of products with *L. monocytogenes* can also originate from the dairy environment, with positive samples often detected on non-food contact surfaces, such as walls and floors, as well as on food contact surfaces [94].

The persistence of *L. monocytogenes* in dairy products and facilities has been documented [27,95], raising concerns about possible contamination along the dairy supply chain, especially in traditional dairy products. The contaminated kajmak samples from our study underline this concern, as these products are made from pasteurized milk, indicating post-pasteurization contamination.

Species other than *L. monocytogenes* were also seen in our samples, with one WBC and four kajmak samples positive for *L. innocua*, and *L. ivanovii* was recovered in one kajmak sample. Similar occurrences of *L. innocua* have been documented in traditional dairy products in Iran [96] and in farm-produced raw milk soft cheese in Portugal [97]. While *L. innocua* are typically considered non-pathogenic, recent studies have described atypical haemolytic virulent strains of *L. innocua* in foods, cautioning about the presence of these species in various foods and the potential implications for food safety and public health [98,99].

When examining hygiene indicators and the presence of *Listeria* spp., *L. monocytogenes* and other *Listeria* spp. were present in 50% of samples where food hygiene standards were not met, mainly due to elevated *E. coli* levels (i.e., above 4 log CFU/g). In addition, yeast and mould counts were significantly higher (*p* < 0.05) in most samples that tested positive for *L. monocytogenes* and other *Listeria* spp. While none of the hygiene and spoilage indicators can be used as a direct indicator for the presence of *Listeria* spp., our data showed that that a large proportion of samples that were contaminated with pathogens also had inadequate levels of hygiene and spoilage microorganisms, indicating a general lack of proper food hygiene practices during production and/or along the short supply chain. This highlights the need for small-scale producers to improve their hygiene and manufacturing practices during their cheese and kajmak production, as well as to provide comprehensive food safety training to mitigate the potential risks of contamination and improve the overall quality and safety of their products [100].

### 3.2. Serogroups of L. monocytogenes Isolates

Using the PCR serotyping method by Doumith et al. [73], *L. monocytogenes* isolates (*n* = 6) were grouped into two serogroups, with the majority of strains (*n* = 4) belonging to group 3 serotypes (1/2b, 3b, and 7) compared to group 1 serotypes (1/2a and 3a) (*n* = 2). Group 3 serotypes are part of lineage I, while group 1 serotypes belong to lineage II. A large study from France has shown that linage I strains have strong association with dairy foods [101]. These results are also in alignment with previous studies that looked at homemade cheeses in Japan [102] and dairy products from Turkey [103], where *L. monocytogenes* 1/2b was a predominant serotype. Since strains belonging to both group 1 and 3 serogroups have caused illnesses before, the presence of these isolates in dairy products in Serbia emphasizes the continued need for vigilance and control measures for *L. monocytogenes* to protect public health.

### 3.3. Antimicrobial Resistance of L. monocytogenes Isolates

All of the tested *L. monocytogenes* (*n* = 6) isolates were sensitive to TET and VAN, and the majority showed sensitivity to GEN (83%), TRS (83%), CTA (50%), and PEN (67%) (Table 4). In the treatment of listeriosis, the therapy of choice is a simultaneous use of β–lactam antibiotics (ampicillin or penicillin G) and an aminoglycoside (gentamicin). Alternatively, a combination of trimethoprim and sulfonamide is used in people who are allergic to penicillin [104,105]. The results of the present study showed that most strains of *L. monocytogenes* isolated from Serbian traditional dairy products are sensitive to PEN and GEN (Table 4). Resistance to TRS has also been seen in other studies [26,106]. Since this antibiotic is used in listeriosis treatment, the finding of TRS resistance in food chain isolates is concerning [107,108,109]. An earlier study from France reported an overall high rate of susceptibility of clinical *L. monocytogenes* strains to a range of antibiotics, including those used in listeriosis treatment [31]. In contrast, varying rates of antibiotic resistance have been reported for foodborne isolates worldwide. In a study conducted in Brazil [109], all of the 50 tested foodborne *L. monocytogenes* strains were sensitive to PEN and GEN, while 68% and 10% were resistant to CLI and TRS, respectively. A high prevalence of the strains sensitive to GEN was also reported in isolates recovered from milk and dairy products from Iran [106]. In the present study, intermediate resistance was observed for CLI (lincosamide; 50%), PEN (33%), and CTA (17%). A higher proportion of isolates possessing resistance or intermediate resistance to CLI and PEN has also been reported by Jorgensen et al. [110], albeit in isolates recovered from produce handling environments.

Notably, all tested strains possessed resistance to OXA (100%). This is similar to findings reported by Harakeh et al. [77], where over 90% of *L. monocytogenes* isolates from dairy products possessed resistance to OXA. This finding is not surprising, since *Listeria* spp. possess natural resistance to many β-lactams due to the lack of penicillin-binding proteins in their bacterial cell membrane [111]. Findings from the present study are also in agreement with many of the other studies that have reported the sensitivity of *L. monocytogenes* strains to VAN [33,77,96,106,112].

TET resistance has been commonly reported in *L. monocytogenes* isolated from food [96,103,106] and is associated with several classes of resistance genes [34]; in the current study, all isolates were susceptible to the antibiotic. This is in line with the findings from an Australian study, which reported sensitivity to TET in 100 *L. monocytogenes* isolates recovered from Australian food chains [113], but it is in contrast to findings from clinical isolates in France, which showed emerging trends for TET resistance [31].

### 3.4. Hygiene and Spoilage Indicator Assessment in Dairy Products

The microbiological evaluation results for the hygiene and spoilage indicators of cheese and kajmak are presented in Table 5.

#### 3.4.1. Coagulase-Positive Staphylococci (CPS) in Dairy Products

The majority of samples in our study had CPS levels below the quantification threshold (Table 5). For raw milk cheeses, 86% of samples met the acceptable microbial quality standards, compared to 98% for pasteurized milk cheeses (Table 6), indicating that post-pasteurization handling may influence CPS levels [114]. Similarly, for kajmak, only 97% of samples were of satisfactory microbial quality, suggesting that the contamination is more likely to be due to post-pasteurization manipulations or cross-contamination than due to contaminated raw milk.

The presence of CPS in food is of concern especially when the amount of CPS exceeds 5 log CFU/g [79,115], as this increases the risk of enterotoxin formation. However, the present study found that 5% of raw milk cheese samples exceeded this threshold, which is consistent with results from Sweden [114] and Belgium [50], which reported 3–10% of samples exceeding the limit. It is important to note that products with inadequate CPS levels also had elevated *E. coli* levels, emphasizing the critical importance of maintaining food hygiene throughout production.

#### 3.4.2. *E. coli* in Dairy Products

The evaluation of *E. coli* levels in cheese is an important indicator of faecal contamination and hygiene practices during cheese production [88,116]. As such, it is included in both the Serbian and European legislation standards [79,115]. Our comprehensive analysis of the cheese samples (Table 5 and Table 6) revealed a varying level of total bacterial contamination, regardless of whether the cheese was made from raw or pasteurized milk. Notably, there was no significant difference between the contamination levels of the two milk types (*p* > 0.05).

*E. coli* has been widely used as an indicator of hygiene in various studies worldwide. For example, Torkar and Teger [91] reported a high bacterial load in soft cheeses, whereas Willis et al. [52] found that 80% of raw milk cheeses met microbiological quality standards for *E. coli*, suggesting a threshold of 2 log CFU/g for acceptability. Previous studies have shown that while raw milk may initially have a higher *E. coli* load, pasteurization effectively eliminates the pathogenic bacteria. However, contamination can occur post-pasteurization due to inadequate heat treatment or poor hygiene during production [114,117]. Our results revealed that 80% and 66% (Table 6) of cheeses from raw and pasteurized milk, respectively, had inadequate bacterial levels. This is consistent with previous results demonstrating a higher prevalence of *E. coli* in raw milk cheeses compared to pasteurized varieties [114]. In Brazil, Fonseca et al. [18] noted that 100% of Minas fresh cheeses failed to meet Brazilian regulatory standards due to *E. coli* counts.

In contrast, 71% of kajmak samples had satisfactory microbiological criteria (Table 6). The highest level of contamination was found in mature kajmak (6.74 log CFU/g), which may be related to hygiene practices during production, since traditional methods often involve manual handling. It is noteworthy that there are no comparable studies on the microbiological quality of kajmak in Serbia, although data are available from other countries [118]. They found that of 20 kajmak samples analysed, 1 sample was unsatisfactory due to the presence of CPS, while 4 samples were classified as unsatisfactory due to the presence of aerobic mesophilic bacteria. However, due to differences in the production process and microbiological profiles, it is difficult to make direct comparisons. Collectively, our findings highlight the need for the continuous monitoring and improvement in hygiene practices in cheese and kajmak production in Serbia to protect consumer health and comply with regulatory standards.

#### 3.4.3. Yeasts and Moulds in Dairy Products

Fermented dairy products, including different cheeses, provide a favourable environment for the survival and competition of yeasts and moulds, in addition to the naturally occurring microbiota [119,120]. While these microorganisms contribute to the unique sensory characteristics of products [121], an excessive presence can lead to spoilage [122]. The present study showed mostly satisfactory levels of yeasts and moulds in the cheese samples (Table 6), although some of them exceeded 8 log CFU/g, indicating potential spoilage. Similar findings in other studies that examined soft cheeses [91] and WBCs [7,14] emphasize the microbial variability between products.

Kajmak samples had a wide range of yeast and mould levels (Table 5), with most meeting microbiological standards. Discrepancies with Turkish kajmak studies suggest that process-related differences may influence microbial quality [17]. However, consistent findings of some products with elevated levels of yeast and mould highlight concerns about suboptimal hygiene in traditional production methods, posing contamination risks during processing and handling.

## 4. Conclusions

In summary, our study found varying levels of microbial contamination, particularly in raw milk cheese and kajmak, highlighting the need for further investigation and action on these products. The presence of *L. monocytogenes* in dairy products after pasteurization underscores the necessity of maintaining proper food hygiene throughout the production process.

The predominance of specific *L. monocytogenes* serogroups and the different antimicrobial resistance patterns emphasize the need for constant surveillance and intervention to mitigate public health risks. Future research should address the virulence potential of the identified serogroups and further investigate the sources and transmission routes of antimicrobial resistance. Overall, this study contributes valuable insights into the microbiological safety and quality of products from small-scale dairy producers in Serbia, emphasizing the need for improved food safety protocols, adherence to hygiene standards, and ongoing monitoring to protect consumer health and ensure regulatory compliance.

## Figures and Tables

**Table 2:** Criteria used for the assessment of the microbiological quality of dairy products.

Dairy Product	Microorganism	Microbiological Quality ^1^			Legal Act/Guide/Literature ^2^
		Satisfactory	Acceptable	Unsatisfactory	
Raw milk cheese	*L. monocytogenes*	Absent in 25 g	N/A ^3^	present in 25 g	[80]
	*Salmonella* spp.	Absent in 25 g	N/A	present in 25 g	[79,80]
	CPS ^4^	≤4	4–5	>5	[79,80]
	*E. coli* ^5^	≤1.30	1.30–2	>2	[52,82]
	Yeasts and moulds	≤6	N/A	>6	[55]
Pasteurized milk cheese	*L. monocytogenes*	Absent in 25 g	N/A	present in 25 g	[79,80]
	*Salmonella* spp.	Absent in 25 g	N/A	present in 25 g	/
	CPS	≤1	N/A	>1	[80]
	*E. coli*	≤2	N/A	>2	[80]
	Yeasts and moulds	≤6	N/A	>6	[55]
Kajmak	*L. monocytogenes*	Absent in 25 g	N/A	present in 25 g	[79]
	*Salmonella* spp.	Absent in 25 g	N/A	present in 25 g	/
	CPS	≤1	N/A	>1	[80]
	*E. coli*	≤3	3–4	>4	[81]
	Yeasts and moulds	≤6	N/A	>6	[55]

^1^ Results are expressed as absent/present in 25 g for the detection methods and as log CFU/g for the quantitative methods. ^2^ Samples were collected in one unit instead of five units as prescribed in legislation [79]. The criteria applied were considered satisfactory, acceptable, and unsatisfactory if the results obtained were below, between, or above the threshold values, respectively. The monitoring legal act [80] specifies that the sampling of one unit of products sold at retail or at the open market is mandatory; the evaluation was carried out in accordance with the Ordinance. ^3^ N/A, not applicable. ^4^ CPS, Coagulase-positive staphylococci. ^5^ Serbian legislation does not set criteria for *E. coli* in raw milk cheeses [79,80]. The criteria on this topic are set for cheeses made from pasteurized milk [79]. This criterion was considered appropriate as it was recommended by the Health Protection Agency [82] and has been used previously to assess this issue in raw milk cheeses [52].

**Table 3:** The overview of the microbiological characteristics of dairy samples contaminated with *Listeria* spp. and *L. monocytogenes* serogroups.

Type of Product and Sample ID	*Listeria* spp. (Detection)	*L. monocytogenes* Serogroups	*E. coli* log CFU/g	CPS ^1^ log CFU/g	Yeast and Moulds log CFU/g
Kajmak, K1	*L. monocytogenes*	Group 3 (1/2b, 3b, 7)	4.08	<1	4.79
Kajmak, K2	*L. monocytogenes*	Group 3 (1/2b, 3b, 7)	<1	<1	2.43
	*L. innocua*	N/A			
Kajmak, K3	*L. monocytogenes*	Group 1 (1/2a, 3a)	3.00	<1	3.08
	*L. ivanovii*	N/A			
Kajmak, K4	*L. monocytogenes*	Group 3 (1/2b, 3b, 7)	<1	<1	3.85
Kajmak, K5	*L. monocytogenes*	Group 3 (1/2b, 3b, 7)	2.81	<1	4.88
Kajmak, K6	*L. innocua*	N/A	4.23	<1	5.00
Kajmak, K7	*L. innocua*	N/A	4.23	<1	4.47
Kajmak, K8	*L. innocua*	N/A	2.60	<1	5.51
Raw milk cheese, C1	*L. monocytogenes*	Group 1 (1/2a, 3a)	4.62	<1	N/A ^2^
Pasteurized milk cheese, C2	*L. innocua*	N/A	4.75	<1	5.48

^1^ CPS, Coagulase-positive staphylococci. ^2^ N/A, not available.

**Table 4:** Antibiotic resistance profile of *L. monocytogenes* (*n* = 6) strains isolated from cheese and kajmak and the percentage of isolates exhibiting each level of antibiotic susceptibility, categorized as resistant, intermediate, and susceptible, relative to the total number of isolates analysed.

Antibiotic	Abbreviation (μg/disc ^1^)	No. of Isolates (%)		
		Resistant	Intermediate	Susceptible
Cefotaxime ^2^	CTA (5 µg)	2 (33)	1 (17)	3 (50)
Clindamycin ^2^	CLI (2 µg)	2 (33)	3 (50)	1 (17)
Gentamicin ^2^	GEN (10 µg)	1 (17)	0	5 (83)
Oxacillin ^2^	OXA (1 µg)	6 (100)	0	0
Penicillin G ^3^	PEN (10 IU)	0	2 (33)	4 (67)
Tetracycline ^2^	TET (30 µg)	0	0	6 (100)
Trimethoprim + Sulfamethoxazole ^3^	TRS (25 µg)	1 (17)	0	5 (83)
Vancomycin ^4^	VAN (5 µg)	0	0	6 (100)

^1^ The concentration of penicillin discs is expressed in international units (IUs). ^2^ CLSI criteria for staphylococci were applied [74,75]. ^3^ EUCAST criteria for *L. monocytogenes* were applied [78]. ^4^ Results were evaluated for *Listeria* spp. in accordance with Dalynn Biologicals (Calgary, AB, Canada).

**Table 5:** Comparison of hygiene and spoilage indicators in dairy products.

Indicators	Raw Milk Cheese			Pasteurized Milk Cheese			Kajmak		
	log CFU/g		<1 log CFU/g	log CFU/g		<1 log CFU/g	log CFU/g		<1 log CFU/g
	*n* (%)	Mean ± SD	*n* (%)	*n*(%)	Mean ± SD	*n* (%)	*n* (%)	Mean ± SD	*n* (%)
CPS ^1^	19 (24) ^a^	4.02 ± 1.49	60 (76)	2 (2) ^b^	3.46 ± 1.49	109 (98)	3 (3)	2.43 ± 0.85	109 (97)
*E. coli*	67 (85)	4.41 ± 1.34	12 (15)	80 (72)	4.12 ± 1.61	31 (28)	60 (54)	3.31 ± 1.41	52 (46)
Yeasts and moulds	77 (98) ^a^	4.99 ± 1.30	2 (2)	111 (100) ^b^	5.29 ± 1.42	0	111 (99)	4.23 ± 1.22	1 (1)

^1^ CPS, Coagulase-positive staphylococci. ^a,b^ Frequencies in the same row marked with superscript letters are significantly different (*p* < 0.05).

**Table 6:** Compliance with microbiological criteria for cheese and kajmak samples.

	Raw Milk Cheese, *n* (%)			Pasteurized Milk Cheese, *n* (%)			Kajmak, *n* (%)		
	CPS ^1^	*E. coli*	Yeast and Moulds	CPS	*E. coli*	Yeast and Moulds	CPS	*E. coli*	Yeast and Moulds
Satisfactory	68 (86)	12 (15)	61 (77)	108 (98)	38 (34)	68 (62)	109 (97)	80 (71)	101 (90)
Acceptable	4 (5)	4 (5)	N/A ^2^	N/A	N/A	N/A	N/A	10 (9)	N/A
Unsatisfactory	7 (9)	63 (80)	18 (23)	2 (2)	73 (66)	42 (38)	3 (3)	22 (20)	11 (10)

^1^ CPS, Coagulase-positive staphylococci. ^2^ N/A, not applicable.

## Data Availability

The original contributions presented in the study are included in the article, further inquiries can be directed to the corresponding author.
